# Contextual influences on physical activity and eating habits -options for action on the community level

**DOI:** 10.1186/s12889-017-4790-x

**Published:** 2017-09-30

**Authors:** Sven Schneider, Katharina Diehl, Tatiana Görig, Laura Schilling, Freia De Bock, Kristina Hoffmann, Maren Albrecht, Diana Sonntag, Joachim Fischer

**Affiliations:** Mannheim Institute for Public Health, Social and Preventive Medicine, Heidelberg University, Ludolf-Krehl-Str. 7-11, D-68167 Mannheim, Germany

**Keywords:** Environment, Exercise, Obesity, Public health, Preventive medicine, Residence characteristics

## Abstract

**Background:**

This conceptual paper aims to illustrate the ways in which communities are able to advance health improvements on a population level. Outcome measures may include increased physical activity and healthier eating habits in particular, as well as an improved health-related quality of life and social cohesion as more generic outcomes.

**Main body:**

The paper begins by asking initial questions: Why did previous health-specific interventions only show moderate effects on an individual level and mixed effects on a population level? What is the added value of a community-based public health perspective compared to the traditional biomedical perspective when it comes to prevention? Why are we living the way we are living? Why do we eat what we eat? Why do we move the way we move?

Subsequently, we illustrate the broad spectrum of contextual interventions available to communities. These can have geographical and technological as well as economic, political, normative and attitude-specific dimensions. It is shown that communities have a strong influence on health-related contexts and decision-making of adults, adolescents and children. In addition contextual characteristics, effects, mediators, moderators and consequences relevant for health can differ greatly between age groups. Both small-scale settings and overarching sectors possess physical, economic, political and sociocultural characteristics that can be proactively influenced by community decision-makers in the sense of a “health in all policies”-strategy.

**Short conclusion:**

After presenting various interdisciplinary approaches to community-based health interventions, the manuscript closes with the following core message: Successful community-based health promotion strategies consist of multilevel – multicomponent interventions on the micro, meso and macro-level-environments.

## Background and aim

Lack of physical activity, poor eating habits, and resultant obesity are spreading endemically worldwide [1, 2]. The obesity epidemic represents a central public health problem on both the individual and societal level in developed and developing countries [3, 4]. Studies, including those involving monozygotic twins, have shown that genetic predisposition plays a large part in determining an individual’s weight and body mass index (BMI), as well as in the development of excess weight gain and obesity [5, 6]. However, the dramatic increase in the prevalence of obesity in the last ten years indicates that other relevant external factors also play a role [7, 8]. In contrast to the stability of our genetic make-up, we have been witnessing dramatic changes in our lifestyles over the last few decades, specifically regarding tertiarization and automation of working environments, mobility and dietary habits [5, 6].

According to current knowledge, excess weight gain is the result of an imbalance between energy intake (e.g. eating habits) and energy use (e.g. physical activity) [3]. For a long time biomedical approaches, pharmaceutical therapies and informative-educational intervention programs (e.g. lifestyle programs and diets) which focused on (re)balancing both these influential factors on an individual level were predominant approaches in excess weight reduction [9, 10]. Although some of these programs were temporarily successful in preventing excess weight gain, the obesity epidemic has so far not been halted [11].

There is broad scientific consensus that the complexity of excess weight is not acknowledged if it is reduced to a problem related only to individual activity and eating habits [2]. Therefore, over the last few years, the biomedical paradigm which focuses on genetic and biological factors has increasingly given way to the Public Health paradigm which focuses on the contexts in which excess weight gain and obesity develop [10, 12]. The concept of obesogenic environments is becoming increasing popular among academics, as well as healthcare policymakers, urban planners, architects and mayors.

This debate paper aims to illustrate the ways in which communities are able to achieve health improvements on a population level using the concept of obesogenic environments by presenting a schematic representation essay, illustrating the concept with the help of an example, and concluding in the challenges of this approach.

## Contextual influences on physical activity and eating habits

### Concept and definition of obesogenic environments

Inspired by the strategies outlined in the Ottawa Charter [13, 14] and Dahlgren and Whitehead’s model of the social determinants of health [15] the concept of obesogenic environments became popular at the end of the 1990s, in particular through the use of the term by Swinburn et al. [4]. Obesogenic environments are defined as the combined influences of surroundings, opportunities or conditions of life on the development of obesity in individuals or populations [4, 16]. According to Hill et al. [17], obesogenic environments foster unhealthy eating habits and physical inactivity. In contrast, the concept of leptogenic environments, another term introduced by Swinburn et al. referring to environments which encourage the achievement and maintenance of a lower bodyweight, has not been well-accepted in the literature [4].

The concept of obesogenic environments includes physical (i.e. geographic and technological), as well as economic, political, and socio-cultural (i.e. normative and attitude-specific) contextual characteristics, that may influence eating habits and physical activity [4, 16].

From the perspective of community actors, it is especially important that obesogenic environments not only influence adults’ weight development, but also childrens´ and adolescents’ [18, 19]. The relevant mechanisms can differ greatly between these age groups: For example, adults often spend time in several, sometimes very diverse and geographically distinct locations (e.g. workplace, home etc.). In contrast, due to their level of dependency (e.g. on socialization agents, institutions and structures) and their restricted mobility and limited own decision-making, children and adolescents are less capable of independently choosing where they spend their time. They are also less able to influence their surroundings or to decide whether to leave a specific environment. In addition, children and adolescents, who are undergoing constant physical and social development, are particularly vulnerable to external influences with regard to their eating and physical exercise habits [16].

### Taxonomy of obesogenic environments

Obesogenic environments can be systematized according to scale, dimension and specific effect [5]. When local environments are the focus of investigation, the scale is referred to as the micro-level. Local environments are also called settings and, for adults, include an individual’s home and workplace, as well as local infrastructures including retail outlets, transportation systems and recreational facilities. In the case of children and adolescents, typical local settings include kindergartens, schools, trade or apprenticeship centers, and sporting clubs in addition to the home and residential environments [4, 16]. Settings are geographically defined, comparatively small and can therefore be fundamentally influenced by local actors (e.g. mayors, politicians, and principals), and those who live and spend time in them [5].

**Fig. 1:**
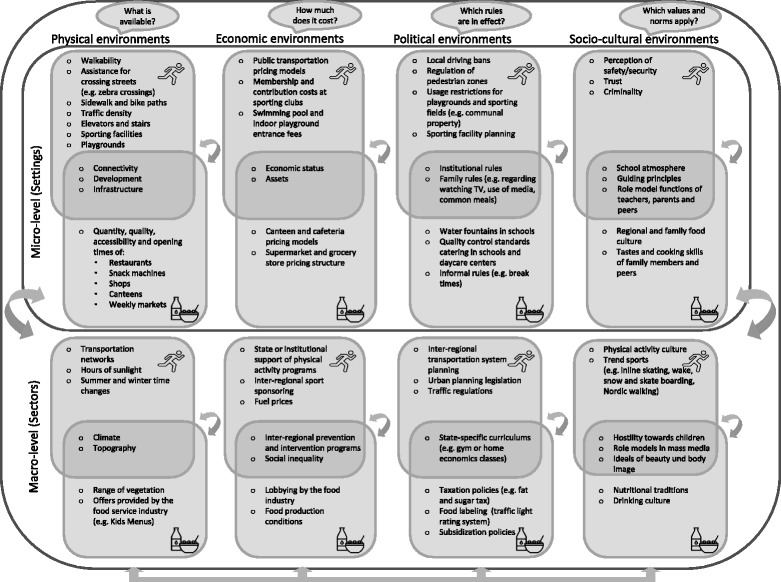
Obesogenic environment –an exemplary systematization of potential factors influencing excess weight gain on the micro and macro-levels (using the Analysis Grid for Environments Linked to Obesity ANGELO [4]). The arrows indicate possible interactions

This micro-level context, also called the micro-environment, is in turn influenced by the macro-level (or macro-environment) [20]. The environments on the macro-level are called sectors. Sectors include, for example, the educational and healthcare systems, the political climate, the mobility and transportation sectors, inter-regional transportation systems, food and sporting industries, and the mass media, as well as the norms, values, and cultures of a given society [4, 16]. This categorization is intended to show that the inter-regional macro-level has an effect on the local micro-level, which in turn has an effect on the lifestyle conditions of individuals. This makes the concept compatible with the social-ecological approach to public health [20]. The taxonomy of environments into settings and sectors is illustrated in Fig. 1. Both settings and sectors have physical, economic, political and socio-cultural dimensions [5]. Figure 1 also shows that specific obesogenic environments can have effects on both eating and physical activity habits. The bidirectional arrows indicate the reciprocal relationships and interactions between these individual levels. The taxonomy outlined here was developed by the Swinburn et al. working group as ANGELO-Framework (ANalysis Grid for Environments Linked to Obesity) [4].

On the local level, the risk of obesity is influenced by an individual’s physical environment, which is in turn influenced by natural and artificial, or constructed, factors. This means that an individual’s physical activity behavior is for instance influenced on the local or micro-level by the traffic density in his or her residential area, by the availability of space to undertake physical activity (green zones, jogging paths, playgrounds, skate-parks, halfpipes etc.) or by the presence of speedlimits, shade, sidewalks and bicycle paths [3, 9, 21, 22].

The food available in regional retail outlets (including weekly markets, restaurants and the foodservice industry), is relevant for individuals’ eating habits [2]. The catering offered at schools is additionally relevant for children and adolescents, and the food offered in work canteens or in restaurants close to workplaces is especially relevant for adults [9, 10, 23–25].

In addition to the physical environment, the economic environment also plays a role in individuals’ physical activity behavior and dietary decision-making (Fig. 1). Examples include swimming pool entrance prices, the cost of public transport, parking fees, membership fees for local sporting clubs and food prices at cafeterias and canteens [2].

The political environment is also relevant in determining individual behaviors in numerous settings (Fig. 1). For children and adolescents such factors may be the restriction of the use of green zones, playgrounds and sporting fields – for example the prohibition of use after a certain hour – and institutional or familial regulations (for example, school rules, community funding policies, or family rules on watching TV, the use of media and common eating times). Factors which are especially relevant for adults include local traffic regulations (for example, access restrictions, pedestrian areas, parking bans) which have an influence on an individual’s choice of modes of transportation [8] (Fig. 1).

Within the settings mentioned, shared values and norms also play a relevant role. These influential factors, which are collectively known as the social-cultural environment, manifest themselves in school and workplace atmospheres, they create social cohesion within neighborhoods and among peer groups and contribute to the subjective feeling of safety in a residential area [2] (Fig. 1).

The physical, economic, political, and socio-cultural environments on the micro-level are influenced by factors on the macro-level. These can be produced by state institutions (such as federal and state governments) or by private enterprises, non-governmental organizations and lobby groups.

Physical facors on a macro-level include the typical inter-regional climate (amount of rainfall, heatwaves), as well as manmade factors such as inter-regionally homogenous menus in catering industry (e.g. “kids’ menu”). An example of an economic factor on a macro-level is the price of gas. This is determined by the world market price as well as national fiscal policy. This amount influences in turn the individual’s decision to use public transport or cycle, instead of driving. Classic political factors on a macro-level include laws (e.g. food legislation) and arbitration (e.g. the planning of transport routes). Socio-cultural factors on a macro-level can for instance be the movement and eating habits of a country.

### Application of the concept of obesogenic environments to an example community

An exemplary scenario may help to illustrate the multifactorial complexity of the concept. Let us imagine a small city in which a large industrial company maintains a production plant. The availability of modern, challenging jobs encourages numerous highly qualified workers with high education level and social status to move to the area over the years. This is combined with a relatively healthy and sustainable dietary culture in comparison with other communities in which more individuals with lower socioeconomic status live (changes to the socio-cultural environment on the micro-level). This healthy dietary culture may lead to the parents in this small town being dissatisfied with the food offered at the local high school cafeteria. The parents start an initiative that, in collaboration with the parents’ association at the school in question, leads to the introduction of quality control standards for the school’s catering services (changes to the political environment on the micro-level). From then on, a civil society foundation initiated by the large industrial company that employs so many of the townspeople subsidizes the cost of school cafeteria meals, which rose as a result of the introduction of the quality control standards, in order to make sure all pupils can afford the food on offer (changes to the economic environment on the micro-level). As a consequence, the pupils are provided with a healthier and more attractive selection of food, (changes to the physical environment on the micro-level). This community-based improvement of what was formerly an obesogenic environment would only move beyond the micro-level if, for example, a lobby group would come together to push for the inter-regional implementation of the new school cafeteria standards. This would require state-wide regulations by the relevant government department (e.g. the Ministry for Cultural Affairs), leading to an extension of the quality control standards to encompass all school cafeterias in the state or country, (changes to the political environment on the macro-level). We have outlined the possibilities and opportunities for societal change as a result of this specific form of lobbying elsewhere [26]. In that paper we also explained how citizen engagement can incorporate the socially disadvantaged and lead to a reduction in health-related societal equality.

### Challenges when investigating obesogenic environments

In sum, the scientific empirical investigation of obesogenic environments is very much in its early stages. Due to the multifactorial etiology of excess weight gain and the complexity of the explanatory model outlined above, analytical studies looking at cultural determinants and evaluating contextual interventions face several key methodological challenges. These will be outlined in the following section:

#### The generalizability of research results for other national contexts

Reviews, which according to our research, are avalailable on the topic of “obesogenic environments”, often focus specifically on demographic groups, or on small subsections of the phenomenon (e.g. physical contextual factors, the food environment, e.g. food industry influences) [5, 18, 19, 22, 25, 27, 28].

Additionally, the aforementioned reviews show that most of the relevant studies were carried out in the USA [9, 18, 24, 28]. This calls into question the generalizability of the results, as the US context cannot be equated directly with for instance the European, Asian or Australian context. For example, historical city structures, some of which have developed over thousands of years, are typical in Europe and Asia and cannot easily be compared to the large-scale, automobile-friendly city structure (urban sprawl) in the USA. Schools in the USA are often located outside the city centers at traffic hubs, whereas schools in Europe are often integrated into downtown areas [23]. The food products on offer also vary greatly between these two societies – both with regard to the rules of production, the range and quality of the products on offer, and the location and ownership structure of foodservice infrastructure [23]. Finally, societal differences in sporting and food culture also have to be taken into account when analyzing data from different countries.

#### Investigating objective and subjective factors

**Fig. 2:**
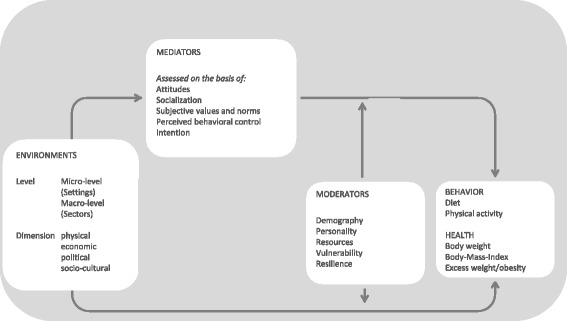
Model of the connection between environmental factors and excess weight gain (based on [26] and [30])

It is possible that objective environmental factors in a certain residential area will be perceived subjectively differently by those living there. Studies involving adults have shown that residents who perceived their surroundings to be subjectively unconducive to physical activity, although this was objectively not the case, undertook less physical activity and had higher BMI-scores than residents whose perceptions where more concurrent with the objective indicators [29]. Children and adolescents’ individual perceptions of residential areas may be influenced by their socialization and by their parents’ norms and values. Kremers et al. [30] characterize these evaluation processes as mediation effects. In addition, moderation effects should be taken into consideration as not every environmental factor has the same effect on every individual. Specific effects are to be expected depending on an individual’s vulnerability, access to resources, gender and age. These methodological considerations are illustrated in a structure chart in Fig. 2. The chart adopts basic principles proposed by Kremers et al. [30] and adapts them to apply to environmental influences on the risk of obesity.

#### Differentiating between compositional and contextual effects

The findings published so far on the interaction between environmental factors and excess weight gain often results from cross-sectional studies, thus impeding causal interpretation. Differences in the prevalence of overweight individuals in an area may not always result from the influence of obesogenic environments. Individual characteristics can also cluster in specific residential areas [31]. When geographic differences in prevalence rates are not caused by environmental factors, but rather are the result of the composition of the population, this is referred to as a compositional effect [26]. For example, when, due to segregation effects in a fictional city, some neighborhoods have a larger proportion of academics than others, the lower prevalence of obesity in those areas could be due to a compositional effect.

The composition of a given population can also be the consequence of individuals selecting where they want to live [12]. Socio-economically better-off families consciously favor certain types of residential areas with good infrastructures. Conversely, socially disadvantaged families are often pushed into more economically deprived neighborhoods with lower rental prices. Selective migration leads to differences in the prevalence of obesity within a city, without the residential environment being the main determinant, per se [32].

#### Consideration of multiple environments in which people spend their time

Furthermore, it should be considered that individuals also leave their local environment [33]. For employees for instance, not only is their immediate neighborhood relevant, but also their workplace environment. On the one hand, this means that local measures do not reach all local inhabitants to the same extent. On the other hand, other individuals who are not residents in the area (guests, commuters, or tourists) may however benefit from these measures.

#### The difficulty of detecting effect through limiting and longterm impacts

From an individual point of view, according to the studies currently available, contextual influence is almost consistently lower than the influence of individual factors [26]. Nevertheless, the perspective presented here is interesting from a preventive point of view, as spatial factors (such as cycle paths, playgrounds, and infrastructures), usually effect demographic groups “around the clock”, long term, and on a broad scale [34]. Benton and his colleagues point out that the detection of the causal effects of changing the built environment in the real world is difficult, because often a before and after measurement is forfeited or no comparition site is considered [35]. Furthermore, the detection of the longterm effects of obesogenic environments have to date been impeded, because most studies are structured in a cross-sectional manner [36].

## Conclusions

Although it is evident that genetic predisposition plays a large part in determining body weight [37], the identification of environment-specific (i.e. contextual) determinants of excess weight gain on the micro and macro-level might be much more important in terms of improving population health. Our daily routines require little physical activity and we have ubiquitous access to cheap, high-energy foods [6]. Although the correlations between contextual factors and weight-related risk factors among the general population identified so far are weak and their explanatory potential may seem low at first glance, we argue that their preventive potential may be substantial, as residential environments have constant and long-term effects not only on individuals, but on entire populations [22, 26, 38]. The concept of the obesogenic environment highlights why battling the obesity epidemic without focused intervention in the living conditions on the micro and macro-levels will continue to be unsuccessful. This increasingly puts the focus on the key role of community actors such as mayors, urban planners, kindergarten and school principals, employers and sports club managers in the promotion of public health.

## Abbreviations

ANGELO: Analysis Grid for Environments Linked to Obesity
BMI: Body Mass Index
